# Potential Errors Resulting from Sex and Age Difference in Assessing Family History of Coronary Heart Disease

**DOI:** 10.2188/jea.14.51

**Published:** 2005-03-18

**Authors:** Tomohiro Saito, Toshihito Furukawa, Seiichiro Nanri, Ikuo Saito

**Affiliations:** 1 Division of Epidemiology, National Institute for Child Health and Development.; 2 Biostatistical Research.; 3 Health Center, Keio University.

**Keywords:** coronary disease, age factors, family, bias (epidemiology), misclassification

## Abstract

BACKGROUND: Coronary heart disease occurs nearly exponentially with age and differently between men and women. Therefore, difference in sex and age of family members yields errors in assessing the family history as a risk factor. The influence of sex and age on the positivity of family history was assessed numerically.

METHODS: Through questionnaires filled in by the parents of 2316 high school students, information was obtained on the past history of coronary heart disease among students’ parents, grandparents, uncles, and aunts. The sex- and age-specific proportion of a positive history was calculated from the past history among the 24,071 family members. The influence of sex and age on a positive history was estimated as odds ratios by logistic regression analysis of the past history.

RESULTS: The odds ratios obtained for sex and age difference were 1.61 (95% confidence interval: 1.42-1.83) and 1.07 (95% confidence interval: 1.06-1.07), respectively. This indicated that a positive history was 1.61 times higher among male members than among female members of the same age, and that a positive history increased by (1.07)^y^, where y was age difference by year.

CONCLUSIONS: Potential errors resulting from disregarding the sex and age of family members can be substantial, judging from the above numerical figures. Some measures to control for the sex and age of family members are required in assessing family history of coronary heart disease.

Sex and age are major risk factors to be considered in epidemiology.^1^ Because morbidity and mortality of coronary heart disease differ between sexes and increase steeply with age,^2^ a control for sex and age of family members should be required in family studies on coronary heart disease.

However, in many studies on the family history of coronary heart disease as a risk factor, this need is neglected or perceived as not very serious: sex and age of family members were not considered at all;^3^^-^^5^ only sex of family members was considered;^6^ or age of family members was considered but their control seemed to be insufficient by labeling children as high-risk with one or more family members who have developed such disease under a certain age.^7^^-^^9^ The last method was insufficient for the following reasons. First, sex of family members was disregarded. Second, in the dichotomous risk evaluation no control for age was exercised below and above the threshold age. Third, the control for age was limited to those who had developed the disease, and no control was made for those who had not yet developed the disease. Those without the disease at age of 50 and 60 years should be assessed differently. There have been studies in which the sex of family members was stratified in addition to the control for age with the above dichotomous risk evaluation.^10^ There exists a study in which age at onset was taken into account.^11^ However, age of those without the disease needs to be also taken into account to improve efficiency in identifying high-risk individuals.

Prevention of coronary heart disease from childhood and youth has been attracting attention worldwide.^12^ In parallel with health education, identifying high-risk children and youth occupies a major portion of the prevention activities. Family history, some have claimed,^13^^,^^14^ is the most important known risk factor at present. Consequently, it is placed as the risk factor of first choice in identifying high-risk children.^7^ Thus, the proper evaluation of family history is crucial.

Under these circumstances, the purpose of this study was three-fold. The first goal is to present the sex- and age-specific proportion of a positive history of coronary heart disease, which would be useful in planning family history studies. This proportion is different from the prevalence of the disease and has been rarely available. The second purpose is to express the influence of sex and age on the positivity of past history numerically for the first time. The third purpose is to call attention to potential errors resulting from disregarding the sex and age of family members.

## METHODS

Family history of coronary heart disease was obtained from a questionnaire survey of family members among 2nd-grade high school students, aged 16 or 17 years, at a high school in Kanagawa Prefecture in 1991, 1993, 1994, and 1995. The questionnaire contained information on the first- and second-degree relatives excluding siblings: parents, grandparents, uncles, and aunts. The collected data included the present age or age at death, and age at onset, by decade, of angina pectoris and myocardial infarction that had been diagnosed or treated by physicians. The questionnaire was handed to 3,145 high school students as part of school health programs and filled in at home by parents. The school health programs have been routinely conducted at the school, but in the study years, health examination, laboratory tests, and the family history questionnaire were added to assess risk for adulthood cerebrovascular and cardiovascular diseases aiming at preventing these diseases from adolescence. Participation to this added health programs was optional. The questionnaire was returned by 2,316 students (a response rate of 73.6%). Two uncle-aunt families were most frequent on both paternal and maternal sides. Among the total of 25,139 family members listed in the returned questionnaire, 24,250 (96.5%) members had full information on the above items asked in the questionnaire. Among the 24,250 members, 179 were below 30 years of age and most of them died prematurely of various causes. These family members were excluded in the following analyses. The reason for exclusion was that the disease targeted in this study was adulthood coronary heart disease caused by multifactorial factors which occurred after age of 30 years. Coronary heart diseases caused by apparent genetic dyslipoproteinemias which might develop below age 30 were out of consideration in this study. Also, in the evaluation of family history of coronary heart disease as a risk factor, family members who were or died below age of 30 years were usually not included in most epidemiologic studies. Thus, the remaining 24,071 family members after the exclusion were used in the analyses.

The 24,071 members were stratified in both sexes into 10-year age intervals either by present age or age at death. In each age interval, the number of those with a positive history of angina pectoris or myocardial infarction and also the number of those who had neither of these disorders were obtained. The age-specific proportion of a positive history for age intervals in each sex was calculated by the following equation: the number with a positive history / (the number with a positive history + the number without a positive history). In quantifying the influence of sex and age on the positivity of past history, which lead to potential errors resulting from disregarding the sex and age of family members, the following logistic regression model was formulated:

log(*p* / (1-*p*)) = intercept + *b*(sex) + *c*(age),

where *p* was the probability of a positive history, and the age was either present age or age at death. From the logistic model, the parameters *b* and *c* were estimated. The odds ratios (ORs) were obtained by an exponent of the estimated *b* or *c* together with its 95% confidence interval by an exponent of [*b* or *c* ± 1.96 × standard error of *b* or *c*]. The interaction between sex and age was also examined, followed by further logistic analyses. The goodness-of-fit of the logistic regression model was assessed by the Hosmer and Lemeshow goodness-of-fit test.^15^

The calculations were performed by the PC-SAS^®^^16^ and the logistic model was carried out by the LOGISTIC procedure.

Informed consent was obtained both from the students and their parents.

## RESULTS

Table 1 shows the age-specific proportion of a positive history of coronary heart disease for 10-year age intervals in both sexes. In the male members the proportion increased nearly exponentially from the youngest age interval to the interval of 60-69 years followed by no increase afterwards. In the female members the proportion increased nearly exponentially until the age interval of 80-89.

**Table 1.** Age-specific proportion of a positive history by sex.

sex	age (year)	No. of subjects	No. of subjects without history	No. of subjects with history (%)	
male	30-39	664	662	2	(0.3)
	40-49	4031	3983	48	(1.2)
	50-59	2754	2651	103	(3.7)
	60-69	1302	1159	143	(11.0)
	70-79	2150	1893	257	(12.0)
	80-89	997	876	121	(12.1)
	90+	79	68	11	(13.9)
	total	11977	11292	685	

female	30-39	600	597	3	(0.5)
	40-49	4608	4584	24	(0.5)
	50-59	2189	2152	37	(1.7)
	60-69	1683	1607	76	(4.5)
	70-79	2311	2119	192	(8.3)
	80-89	677	599	78	(11.5)
	90+	26	25	1	(3.9)
	total	12094	11683	411	

The influence of sex and age on a positive history estimated by the logistic regression analysis was the following. The OR for sex difference was 1.61 with a 95% confidence interval (CI) of 1.42-1.83. This means that a positive history of coronary heart disease was 1.61 times higher in male family members than in female family members of the same age. The OR for age difference was 1.07 with a 95% CI of 1.06-1.07. This means that a positive history of coronary heart disease increased by 1.07 with an increase of age of family members by one year. ORs resulting from age differences of y years were obtained by (1.07)^y^. The OR for a 5-year difference was 1.38, and for a 10-year difference it was 1.91.

In the logistic regression analysis including an interaction term between sex and age, the interaction became statistically significant (p<0.05). The result of the Hosmer-Lemeshow goodness-of-fit test was also statistically significant (p<0.001). Therefore, logistic regression analysis was done separately in each sex and age interval. The results are shown in Table 2. The logistic regression analysis was done separately for the new 4 intervals made up of two, or three in the youngest and oldest interval, adjoining 10-year age intervals. For each new age interval, the logistic regression model including only the age variable was carried out in the male and female groups, and these results are shown in the rows titled “From age difference.” The logistic regression model including sex and age variables was also carried out and these results are shown in the rows titled “From sex difference.” The results of the Hosmer-Lemeshow goodness-of-fit test for the model including both sex and age were also carried out. Interaction between sex and age was evaluated in each age interval.

**Table 2.** The influence of sex and age on a positive history expressed by odds ratio obtained from logistic analysis of family history.

		age (year) *			

		30-59	50-69	60-79	70-99
From age difference ^†^	Male	1.15 (1.12-1.19)	1.09 (1.07-1.11)	1.02 (0.999-1.04)	1.01 (0.99-1.03)
	Female	1.12 (1.08-1.17)	1.09 (1.06-1.12)	1.09 (1.06-1.12)	1.04 (1.01-1.06)

From sex difference ^‡^		2.22 (1.67-2.95)	2.51 (2.02-3.12)	1.73 (1.48-2.02)	1.35 (1.14-1.59)

Goodness-of-fit (p) ^§^		0.61	0.18	0.63	0.00

Interaction between sex and age ^||^		1.03 (0.98-1.08)	1.00 (0.97-1.04)	0.93 (0.91-0.96)	0.97 (0.94-1.00)

Ninty-five percent confidence intervals in parentheses.

* : The age interval was an interval extending over 2 or 3 adjacent 10-year age intervals.

† : Odds ratios from logistic analysis including only age variable.

‡ : Odds ratios from logistic analysis including both sex and age variables.

§ : Hosmer-Lemeshow goodness-of-fit test.

|| :Odds ratios of the interaction term in the logistic analysis.

The ORs for age difference in male members were small in the age intervals of 60-79 and 70-99, and they were not statistically significant (p>0.05), including unity in their 95% CIs. The ORs in female members tended to decrease with the advancement of age. However, they were fairly large in terms of the influence of age difference on the positivity of the history, and all the ORs were statistically significant (P<0.05). The OR for sex difference was largest in the age interval of 50-69, followed by the age intervals of 30-59, 60-79, and 70-99 in the order of magnitude. All the ORs for sex difference were statistically significant (p<0.05) with the 95% confidence intervals above unity. The Hosmer-Lemeshow goodness-of-fit test was statistically significant (P<0.05) only in the age interval of 70-99, and the logistic regression model including sex and age was justified in the other age intervals.

The interaction between sex and age was significant (p<0.05) only in the age interval of 60-79. Its 95% CI did not include unity while in the other age intervals the 95% CIs included unity. The interaction OR below unity meant that the sex difference became smaller with the increase of age, and that above unity meant that the sex difference became larger with the increase of age. The logistic regression analysis including sex and age variables was justifiable with the exception of the interval 60-79, although the influence of age difference on a positive history was evaluated separately in each sex and their results are shown in the table. The ORs from age difference and the 95% CIs for the 4 age intervals obtained in the logistic regression analysis including sex and age variables were 1.14 (95% CI: 1.12-1.17), 1.09 (95% CI: 1.07-1.11), 1.04 (95% CI: 1.03-1.06), and 1.02 (95% CI: 1.00-1.03). The ORs in the table can be regarded as more precise estimates, but these estimates in practical use are also justifiable except for the age interval of 60-79. The interpretation of the ORs is the same in principle as that already described in the analysis for the whole age interval.

To summarize the results in Table 2, the influence of age difference on a positive history was not small below age of 70 years in males and throughout life in females. The influence of sex difference on a positive history was not small throughout the life, though the influence became smaller with age after 60 years. The male surplus in a positive history increased until the 60s followed by a decrease afterwards. The results from the logistic regression analysis should be justifiable except for ages above the 70s.

A closer look at the age-specific proportion of a positive history and the detailed analysis provided different ORs for age difference and sex difference. The ORs of 1.61 for sex difference and 1.07 for age difference in the whole age interval stood as summary statistics.

## DISCUSSION

This paper dealt with sex-specific and age-specific proportion of a positive family history and not with incidence or prevalence. Therefore, the ORs for sex and age difference in this study are applicable to the evaluation of family history and not applicable to incidence or prevalence data. In other words, the ORs do not represent sex- or age-specific risk of individuals.

The inadequacy in assessing family history of coronary heart disease by a method which neglects sex and age of family members is apparent. The difference in the proportion of a positive history between male and female family members as seen in Table 1 is not small. The risk for a person whose father, uncles or grandfathers has a positive history and a risk for a person whose mother, aunts or grandmothers has a positive history should be different. The latter should be given a higher risk. A woman who developed the disease at a certain age is regarded as carrying a heavier risk for the disease than a man who developed the disease at the same age. This is a strong message gathered from the results of this study.

The results of the logistic regression analysis also indicated that the magnitude of errors resulting from disregarding the sex and age of family members could be substantial. A positive history of coronary heart disease in male family members is, roughly speaking, expected to be 1.6 times higher than that in female family members of the same age. If the age difference between members of compared families is larger than 5 years, a probability of having a family member with the disease increases, on the average, by 1.38 times, and this can be of the same magnitude as the risk ratios of other risk factors.^17^ Therefore, to assess risk factors among children and youth of adulthood coronary disease from family history, evaluation needs to be done separately in both sexes and the control for age among families may need to be less than 5 years.

The degree of actual bias or misclassification depends on the study type and the genetic model working in this disorder. The results would be biased in a case-control study if the sex and age of family members are disregarded.^18^ The results would be diluted towards no association due to misclassification in a cohort study.^18^ The degree of actual bias or misclassification in a study cannot be accurately assessed because the genetic model, possibly a multifactorial inheritance, has not been clearly determined yet. However, it is likely that the genetic role in coronary heart disease is not of a threshold type with age but more of a continuous type. Possible uses of the results of this study under this uncertainty are the following; in a particular study in which a genetic model is assumed, a possible bias or misclassification will be roughly speculated using the method or results of this study. The numerical figures obtained in this study should be of some use for this purpose.

There remains the question of the validity of the questionnaire survey: whether the information on present age, age at death, the past history of coronary heart disease and age at onset by decade was reliable. Since the questionnaire was filled in at home by parents, present age or age at death must be quite reliable. It is unlikely that parents did not know the present age or age at death of their brothers, sisters, and parents – the uncles, aunts, and grandparents in the questionnaire. It is also unlikely that parents were mistaken in answering the past history of coronary heart disease and its onset by decade age among their brothers and sisters. They were mostly in their 40s and 50s, and members of average families contact and meet each other regularly once or twice a year in Japan. The grandparents in the questionnaire were mostly in their 60s and 70s by present age or age at death. Their sons and daughters – the parents in the questionnaire – are likely to know the past history and its onset by decade age of such serious diseases as angina pectoris and myocardial infarction in their parents. In this connection it is stated that the agreement between questionnaire data and medical records in middle-aged and elderly men and women was reasonably good for cardiovascular diseases;^19^ disagreement was not so serious as to jeopardize the results of this study.

For the purpose of evaluating the precision of the questionnaire employed here, we conducted a study in which agreement was examined between two questionnaire surveys, administered twice among the same study subjects with a one-year interval.^20^ The proportion of contradicting answers - namely an interchange between the presence and absence of the past history - between the two surveys among parents and grandparents were 0.5% and 3.4%, respectively. The proportion of contradicting answers regarding age at onset was 0.1% among parents and 1.3% among grandparents.

Given that the information on the past history was not quite accurate among grandparents, its effect on the estimated OR should be small in the logistic model used. For example, a 10% under-reporting, in this study, of a positive history in the age group of the 60s and above yields a revised OR from age difference within 1% of the original estimate: not large enough to endanger the results and the interpretation obtained in this study. Generally speaking, the model used in this study is robust to possible inaccuracies in the age-specific proportion of a positive history.

An age difference as large as 5 years between parents of different families is not infrequent, judged from the distribution of age at marriage and the age of mothers at delivery.^21^ In assessing family history of coronary heart disease as a risk factor, some measures should be taken into control for age of family members in both sexes. The measures may be stratification, matching, multivariate analysis or the use of some risk indices which control for age.

Risk was known to differ depending on various definitions of positive family history.^22^ In this context, a risk index which controls for sex and age was proposed for coronary heart disease.^23^^,^^24^ In this method, a family risk score was calculated based on the difference between the observed number of affected family members and the expected number calculated from the sex- and age-specific cumulative incidence in a general population. In another study, an index of familial predisposition was developed.^25^ All relevant family parameters including sex and age were employed and weighted in the statistical model according to their relative importance. A quantitative method was proposed using age of family members or age at onset as a parameter and this method seemed to be useful.^26^ Another quantitative method was proposed in which an incidence rate of the disease among family members was used.^18^ In a study on diabetes mellitus the frequency of diabetes in a family was adjusted by considering the age of family members.^27^ This was done by giving to each family member an age-dependent probability of developing the disease, based on empirical data of age at onset of the disease, under the postulation that all the family members would reach the assumed maximal age of 85 years. In another study of diabetes mellitus the fasting glucose levels of family members were adjusted by sex and age, and these adjusted levels were used as a genetic index instead of a positive history.^28^ Also in diabetes mellitus, the probable risk of diabetes in the offspring was computed using the Kaplan-Meier product limit estimate.^29^ However, in that study the estimate was for the offspring as a whole and not for an individual offspring in a particular family. Use of these methods depends on the type of study and availability of data.

Failure to employ any measures will result in bias or misclassification. The bias may be differential, which exaggerates or underestimates the familial load.^30^ An example of exaggeration would be a case-control study in which family history is compared, without matching for age, between patients of coronary heart disease and controls. Because the incidence of coronary heart disease increases with age, it is likely, without any control, that the patients’ age will be greater than the controls’ age. It follows naturally that the first-degree relatives of the patients will be older, on the average, than those of the controls. For this reason only, a positive history among the relatives will be more frequent among the patients than among the controls. In a cohort study the effect may be misclassification. Individuals may be labeled as high-risk because of the old age of family members. In this case the association is diluted toward the null condition.^30^ This will lead to a weaker association or no association between a positive family history and occurrence of coronary heart disease. The bias or misclassification is expected to be greater in Western countries than in Japan. In most Western countries the incidence and mortality from coronary heart disease increase more steeply with age than they do in Japan.^2^ Therefore, the proportion of a positive history also increases more steeply with age than those in Table 1, resulting in higher ORs than those obtained in this study.

The necessity for considering sex and age in assessing family history and the degree of the potential bias or misclassification need to be borne in mind also in clinical practice, in which family history is almost always taken. The possibility of such error must be carefully examined if family history is taken as a risk factor for a patient.

Lack of control for the sex and age of family members is not uncommon in evaluating family history of other diseases with a steep increase in age-specific incidence including hypertension,^31^ diabetes,^32^ and stroke.^33^ Potential bias or misclassification and the need for a control for sex and age in family history assessment need to be widely recognized.
